# Comparative Analysis of the Evolution of Green Leaf Volatiles and Aroma in Six *Vitis vinifera* L. Cultivars during Berry Maturation in the Chinese Loess Plateau Region

**DOI:** 10.3390/foods13081207

**Published:** 2024-04-16

**Authors:** Huawei Chen, Zhenwen Zhang, Lijian Zhang, Shijian Bai, Pengfei Ning, Shichao Wei, Sha Xie, Qingqing Zeng

**Affiliations:** 1 College of Enology, Northwest A&F University, No. 22 Xinong Road, Yangling 712100, China; 15699338225@163.com (H.C.); zhangzhw60@nwsuaf.edu.cn (Z.Z.); 18379236260@163.com (L.Z.); wsc950323@163.com (S.W.); 2 Xinjiang Uighur Autonomous Reg Grapes & Melons Research, Turpan 838000, China; bsj19861103@163.com; 3 Yaojing Winery Co., Ltd., Linfen 041500, China; ningpengfei1985@163.com; 4 Shaanxi Engineering Research Center for Viti-Viniculture, Yangling 712100, China

**Keywords:** wine grapes, loess plateau, green leaf volatiles, LOX-HPL pathway

## Abstract

Green leaf volatiles (GLVs) are important in giving grape a fresh and green aroma. But the changes in GLVs during the phenological development of grapevines are not well known. This study analyzed the GLVs and transcription levels of associated biosynthetic genes in six grape species from the Loess Plateau region at five stages of maturation. Thirteen GLVs were detected, showing unique patterns for each grape type at various growth phases. The primary components in six grapes were (E)-2-hexenal, (E)-2-hexen-1-ol, and hexanal. With the exception of Cabernet Franc in 2019, the overall GLV contents of the six types generally increased during growth and development, peaking or stabilizing at harvest. And Sauvignon Blanc, Cabernet Gernischt, and Cabernet Sauvignon exhibited higher total contents among the varieties. PLS-DA analysis revealed 3-hexenal’s high VIP scores across two years, underscoring its critical role in grape variety classification. Correlation analysis revealed a strong positive correlation between the levels of hexanal, 1-hexanol, (E)-2-hexen-1-ol, (Z)-3-hexenyl acetate, nonanal, and (E, E)-2,6-nonadienal and the expression of *VvHPL* and *VvAAT* genes in the LOX-HPL pathway. Specifically, *VvHPL* emerges as a potential candidate gene responsible for species–specific differences in GLV compounds. Comprehending the changing patterns in the biosynthesis and accumulation of GLVs offers viticulturists and enologists the opportunity to devise targeted strategies for improving the aromatic profile of grapes and wines.

## 1. Introduction

Volatile substances are important secondary metabolites in grapes that are essential for the sensory assessment of grapes and wines [1,2]. The aroma components derived from grapes mainly include C13-norisoprene, terpenes, C6/C9 compounds, methoxypyrazines, and thiol substances, among others [3]. Among them, C6/C9 compounds are present in elevated levels in grapes and wines, with concentrations reaching mg L^−1^ levels, and are synthesized through the pathway of lipoxygenase from precursor fatty acids [4]. Additionally, C6/C9 components consist of C6 and C9 aldehydes, alcohols, and esters, which are significant fragrance constituents found in grapes and wines. C6/C9 components, sometimes referred to as green leaf volatiles (GLVs), are emitted by plants as signaling molecules during times of environmental stress, aiding in the transmission of signals [5]. GLVs in grapes and wines have a low olfactory threshold and are characterized by green, fresh, fruity, and floral aromas; they play a significant role in providing aroma in certain grape varietals used in winemaking [6,7].

The lipoxygenase-hydroperoxide lyase (LOX-HPL) pathway facilitates the direct production of C6 and C9 aldehydes by the oxidative cleavage of linoleic and α-linolenic acids [8]. The primary enzymes found in grape berries within this system are Lipoxygenase (LOX), hydroperoxide lyase (HPL), alcohol dehydrogenase (ADH), and acyltransferase (AAT) [9]. Previous research has demonstrated that the biosynthesis and accumulation of GLVs in grapes are influenced through vineyard management techniques such as leaf removal [10], plant development regulation [11,12], irrigation [13], and foliar fertilization [14], and vary with grape variety and stage of berry maturation [15]. Moreover, a number of studies have investigated the fluctuating levels of volatile compounds as grape berries mature [16]. Prior research has primarily examined individual grape varieties or viticultural methods, with less research initiatives evaluating the developmental processes of various varieties, particularly with the dynamics of GLVs [17]. Investigations into the the terpenoid components in Muscat Hamburg and Sauvignon Blanc vary at different maturation stages [6,18]. Concurrently, another study found variations in volatile esters between Cabernet Sauvignon and Syrah grapes and wines. It suggests that the existence of a more active LOX-HPL pathway in Syrah grapes affects esters the most [19]. Thus, the importance of C6 substances in grapes has been substantiated with regard to varietal typicity, and the distinct variations in the LOX-HPL pathway have garnered increased attention. Nonetheless, the progression of GLV profiles and the expression of LOX-HPL pathway genes during berry development in various grape species have not been fully elucidated.

As a rapidly developing wine-producing powerhouse, China has established a series of unique grape-growing regions. The Loess Plateau area, located in the central section of the Yellow River in China, is particularly notable. The microclimates easily formed in this distinctive plateau region significantly impact the quality attributes of grapes and wines [20]. The Loess Plateau in China is well-suited for grape growth. It supports key wine grape varieties such as Chardonnay, Sauvignon Blanc, Cabernet Sauvignon, Cabernet Franc, Cabernet Gernischt, and Marselan. To better explore the biosynthesis and accumulation mechanisms of GLVs in grapes, the present study investigated the GLV profile during the ripening process of six widely planted wine grape varieties in the Loess Plateau area, revealing variations in GLV profiles across grape varieties and developmental stages. Furthermore, the present research thoroughly examines how genes in the LOX-HPL pathway are expressed at five different developmental phases in the berries of six types, analyzing their links with GLV metabolism and regulation. The present study highlights the dynamic shifts in GLV components across the developmental stages of fruit in various grape varieties. It establishes a crucial foundation for identifying the development of distinctive aromas in the grapes and wines extensively cultivated in the Loess Plateau region.

## 2. Materials and Methods

### 2.1. Field Conditions and Materials

Grape materials, including Sauvignon Blanc, Chardonnay, Cabernet Franc, Cabernet Sauvignon, Cabernet Gernischt and Marselan were sampled in 2019 and 2020 from the Yaojing vineyard in Linfen city, Shanxi Province, China. The vineyard management procedures, including irrigation and fertilization, adhered to local regulations. The grape planting density was 0.5 × 3 m. All the samples were randomly collected at the following stages: E-L 34 (berries begin to soften; Brix starts to increase); E-L 35 (berries begin to develop colour and become larger); E-L 36 (berries exhibit medium Brix values); E-L 37 (berries are not fully mature); and E-L 38 (berries are harvested at maturity). The E-L system was developed according to Coombe et al. (1995) [21]. Each replication involved sampling around 500 berries, with each type being evaluated three times. The samples were immediately placed on dry ice and stored in a −80 °C freezer for future analysis.

### 2.2. Analysis of the Chemical Characteristics of Grapes

The grapes’ chemical properties, including total soluble solid content, titratable acidity, and pH, were analyzed following the method outlined by Ju et al. [9].

### 2.3. Determination of Meteorological Data

Meteorological data for the years 2019 and 2020, encompassing temperatures, rainfall, along with hours of sunshine, were acquired from the China Meteorological Data Service Center (https://data.cma.cn/en (accessed on 10 December 2020)) (Table S1).

### 2.4. Analysis of GLV Compounds in Grapes by GC-MS

GLV components were isolated from grapes using the method as described by Yue et al. [12]. In total, 50 g of grapes, frozen with liquid nitrogen, were placed in a mortar, after which the seeds were removed and mixed with polyvinylpolypyrrolidone and D-(+) gluconic acid δ-lactone. Five milliliters of the supernatant were transferred to sample bottle, to which 10 μL of 4-methyl-2-pentanol and 1.00 g of NaCl. This setup was then subjected to heating and stirring at 40 °C for 30 min on a magnetic-stirrer-heating plate. Post-heating, we placed the extraction head into the vial’s headspace for 30 min, subsequently attaching it to the injection port of the gas chromatograph for the adsorption step.

For the analysis of Green Leaf Volatile (GLV) compounds, we employed an Agilent 7890B Gas Chromatograph coupled with an Agilent Mass Spectrometer and used an HP-INNOWAX column (dimensions: 60 m × 0.25 mm × 0.25 µm). The carrier gas, helium, was maintained at a flow rate of 1 mL/minute. We set the mass spectrometer (MS) inlet temperature at 250 °C. The temperature program for the oven started at 50 °C, held for one minute, then increased to 220 °C at a rate of 3 °C/minute, where it was maintained for 5 min. The mass spectrometer was operated in the Electron Ionization (EI) mode, with an ion source temperature of 250 °C, electron energy at 70 eV, and the mass scan ranging from 30 to 350 u. We performed the analysis in triplicate for each sample.

Identification of the GLV compounds was achieved by comparing their retention times with those of authentic standards and utilizing the National Institute of Standards and Technology Library (NIST 11) for reference. For quantitative analysis, we applied standard curves of the identified compounds (Table S9).

### 2.5. Odor Activity Values

The impact of GLVs on the distinct aroma of grapes was assessed quantitatively by the odor activity value (OAV), citing previous research on the descriptive vocabulary of aroma compounds and their odor thresholds [22].

### 2.6. Expression of LOX-HPL Pathway Genes

The extraction of total RNA from samples was carried out following the methodology of a previous study [1]. In total, 1 μg of purified RNA was utilized, and cDNA was generated through reverse transcription with the TransGen cDNA Synthesis Kit (Beijing, China). Gene expression levels were measured using quantitative real-time PCR (qRT-PCR), with each sample analyzed in triplicate. VviActin served as the reference gene. Specific primers used for the LOX-HPL pathway in this study are listed in Table S10. Gene expression levels were quantified using the 2^−ΔΔCT^ method [23].

### 2.7. Statistical Analysis

All experimental data were analyzed using SPSS 20.0. Statistical treatments, including Analysis of Variance (ANOVA) and Tukey’s test, were applied (*p* < 0.05). Histograms were prepared using Origin 2021. Heatmaps were drawn using ChiPlot v2.1 (https://www.chiplot.online (accessed on 9 November 2023)).

## 3. Results and Discussion

### 3.1. Primary Chemical Characteristics of Berries

The chemical characteristics of six grape varieties were measured at harvest (Table S2). In 2019, the soluble solids of the six varieties ranged from 19.22 to 23.65 °Brix. In 2020, the soluble solids of the six varieties ranged between 18.87 and 21.53 °Brix. Marselan had higher levels of soluble solids in the grapes across both years, while Chardonnay had lower ones. Chardonnay and Sauvignon Blanc had titratable acidity levels between 5.47 and 6.40 g L^−1^ in both years, which were significantly greater than those of the other four varieties. This elevated acidity is crucial for the freshness and longevity of wines produced from these grapes. The pH values of the grape berry from the different varieties studied ranged from 3.80 to 4.31 over the two years, reflecting the intrinsic balance between acidity and basicity that influences wine stability and color.

GLVs, known for their contribution to the fresh, herbaceous aromas in wines, are intricately related to the grape’s chemical composition [17]. The higher acidity in Chardonnay and Sauvignon Blanc, for instance, not only underscores their freshness but also potentially enhances the expression of GLVs, contributing to the distinct varietal aromas that define these wines. Conversely, the variation in soluble solids and pH levels across the varieties suggests a complex influence on the synthesis and perception of GLVs, further impacting the aromatic complexity of grapes and wines.

### 3.2. Green Leaf Volatile Evolution

**Figure 1:**
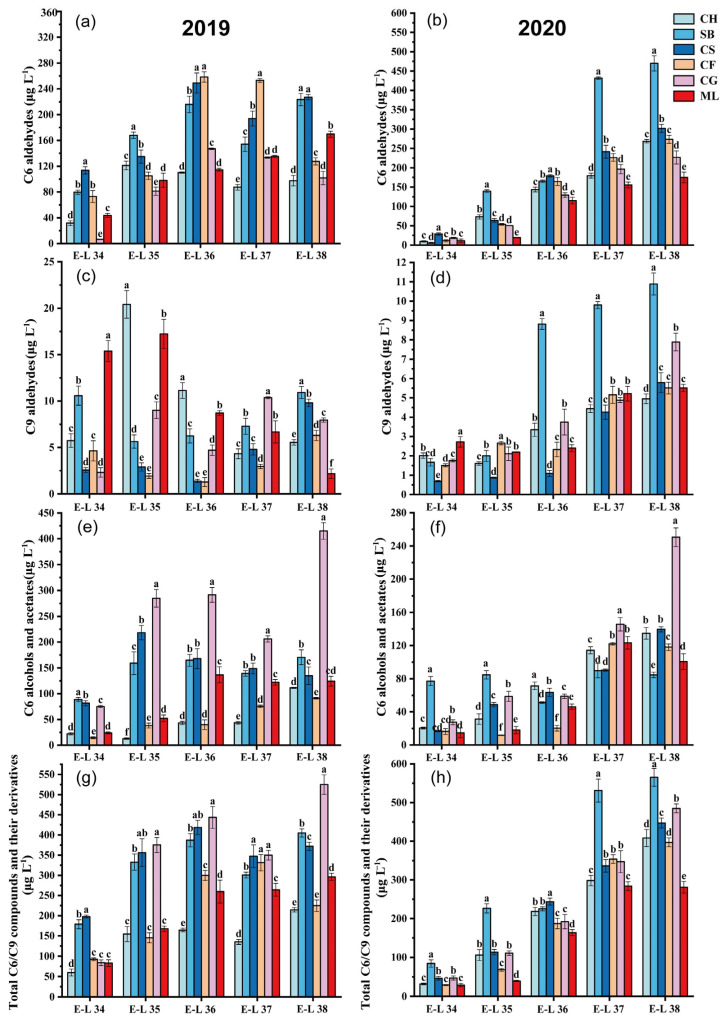
Analysis of GLVs in six grape types during the 2019 and 2020 vintages. CH, Chardonnay; SB, Sauvignon Blanc; CS, Cabernet Sauvignon; CF, Cabernet Franc; CG, Cabernet Gernischt; ML, Marselan. (**a**) 2019 C6 aldehydes; (**b**) 2020 C6 aldehydes; (**c**) 2019 C9 aldehydes; (**d**) 2020 C9 aldehydes; (**e**) 2019 C6 alcohols and acetates; (**f**) 2020 C6 alcohols and acetates; (**g**) 2019 Total C6/C9 compounds and their derivatives; (**h**) 2020 Total C6/C9 compounds and their derivatives. Different letters show significant differences between treatments by Tukey’s test (*p* ≤ 0.05) (Consult the online version of this article for an elucidation of the color codes mentioned in the figure legend).

GLVs are generated from unsaturated fatty acids through enzymatic processes involving lipoxygenases, hydroperoxide lyases, as well as alcohol dehydrogenases [24]. GLVs in grapes are responsible for citrus, leafy, and green sensory attributes, and they play a significant role in the fragrance profile of grapes [25]. We studied the development of total GLVs in six grape types, as shown in Figure 1a–h.

In 2019, during the grape maturation process, all the varieties exhibited lower C6 aldehyde concentration at E-L 34 (Figure 1a). The C6 aldehyde concentration in the six grape types at harvest varied from 97.59 to 227.11 μg L^−1^. Cabernet Sauvignon and Sauvignon Blanc had significantly greater total C6 aldehyde levels at harvest E-L38 than the other varieties. The C6 aldehyde growth pattern in the 2020 grapes resembled that of 2019, showing lower concentrations in the early stages of development and steady accumulation with maturation (Figure 1b). Compared with those of the other varieties, the overall C6 aldehyde levels in Sauvignon Blanc and Cabernet Sauvignon significantly increased throughout development. The C6 aldehyde levels in the six grape types at harvest in 2020 varied between 174.93 and 469.86 μg L^−1^. In line with our results, Lu et al. [26] found that C6 aldehyde concentrations were different in summer and winter, suggesting that the yearly variation in C6 aldehyde levels in grapes might be due to seasonal weather differences, necessitating additional research.

In 2019, the trends in total C6 alcohols and acetates varied significantly among different types (Figure 1c). The concentrations of C6 alcohol and acetate in grapes rose by factors of 4.04—(Chardonnay), 0.93—(Sauvignon Blanc), 0.65—(Cabernet Sauvignon), 5.25—(Cabernet Franc), 4.52—(Cabernet Gernischt), and 4.18—fold (Marselan). In 2020, Sauvignon Blanc grapes exhibited the greatest levels of total C6 alcohol and acetate concentrations at E-L 34 and E-L 35. The highest levels of total C6 alcohols and ethyl esters at harvest were discovered in Cabernet Gernischt grapes, with Cabernet Sauvignon and Merlot varietals displaying lower levels (Figure 1d). The growth trends of total C6 alcohol and acetate varied within the six grape types in 2020 compared to 2019. Except for those of Chardonnay and Cabernet Franc, the total C6 alcohol and acetate contents of the 2020 grapes were significantly lower than those of the 2019 harvest, which was possibly related to climate differences. A warmer climate facilitates the transformation of free C6 aldehydes into C6 alcohols, which generally have higher herbaceous odor thresholds (negative aroma) than the corresponding aldehydes [26]. Additionally, alcohols are more likely to form esters, thereby minimizing the herbaceous characteristics of aldehydes and maximizing the fruity characteristics of esters [25]. 

C9 compounds in grapes primarily exist in aldehyde form, such as (E)-2-nonenal, (E, E)-2,6-nonadienal and nonanal. C9 aldehydes, even in low quantities, have low thresholds that enable them to impart citrus and cucumber scents to grapes [13]. In 2019, Marselan at E-L 34 exhibited the highest C9 aldehyde level compared to all other grape types (Figure 1e). During the mature stage (E-L 38), Sauvignon Blanc exhibited the highest concentration of C9 aldehyde, with Cabernet Sauvignon following closely behind. The overall accumulation trend of C9 aldehydes in 2020 was similar to that in the 2019 vintage, with six varieties gradually accumulating C9 aldehydes or exhibiting stabilization of C9 aldehyde levels with growth and development (Figure 1f). A comparison with existing literature reveals that the presence and concentration of C9 aldehydes in grapes are influenced by a multitude of factors, including genetic varietal characteristics, viticultural practices, and, notably, climatic conditions [27,28]. Studies have highlighted the sensitivity of C9 aldehyde synthesis to environmental factors, suggesting that variations in temperature, sunlight exposure, and rainfall can significantly affect their accumulation in grapes [29]. The meteorological data presented for the vintages 2019 and 2020 (Table S1) provide critical insights into the climatic backdrop against which these aromatic compounds developed. The 2019 vintage, characterized by higher mean maximum temperatures and lower relative humidity during critical growth phases, might have facilitated the enhanced synthesis or preservation of C9 aldehydes in Marselan and, subsequently, Sauvignon Blanc. Conversely, the 2020 vintage, with its increased rainfall and slightly lower temperatures, suggests a climatic influence on the slightly different accumulation pattern observed.

With respect to the total GLV contents in 2019, except for Cabernet Franc, in which the GLV contents slightly declined at maturity, all the other varieties exhibited maximum GLV levels at maturity (Figure 1g). The GLV contents of all the varieties at maturity ranged from 214.86 to 524.81 μg L^−1^. Cabernet Gernischt and Cabernet Sauvignon had higher GLV contents during growth and development. At harvest, Cabernet Gernischt exhibited the greatest GLV contents. The GLV concentration among all berries at harvest in the 2020 vintage varied between 281.14 and 565.31 μg L^−1^ (Figure 1h). Sauvignon Blanc and Cabernet Gernischt had higher total GLV contents at harvest, while Marselan and Chardonnay had lower GLV contents at harvest.

As all grape samples originated from the same vineyard, differences in external influences were reduced when comparing different grape kinds and vintages. These results indicate that variations in GLV contents among different varieties might be due to genetic variations affecting LOX-HPL gene expression and the influence of different environmental conditions [13] (Wang et al., 2019). Consistent with our findings, Yue et al. [12] found that the vintage had an impact on the variation patterns of GLVs. Seasonal variations in the microclimate, such as sunlight and rainfall, could impact gene expression in the GLVs synthesis pathway, thereby affecting the function of enzymes associated with GLVs biosynthesis [8,18]. The cultivar significantly impacts the aroma of grapes. Li et al. [1] noted substantial variations in GLV levels between Marselan and Merlot scion–rootstock grapes. There is insufficient research on the alterations in GLV compounds associated with green aromas as different wine grape varieties ripen. Our study fills this gap by demonstrating that GLV volatiles not only affect the aromatic characteristics of grapes but are also a component of varietal typicity.

### 3.3. Analysis of Green Leaf Volatile Profiles in Grapes as They Mature

**Figure 2:**
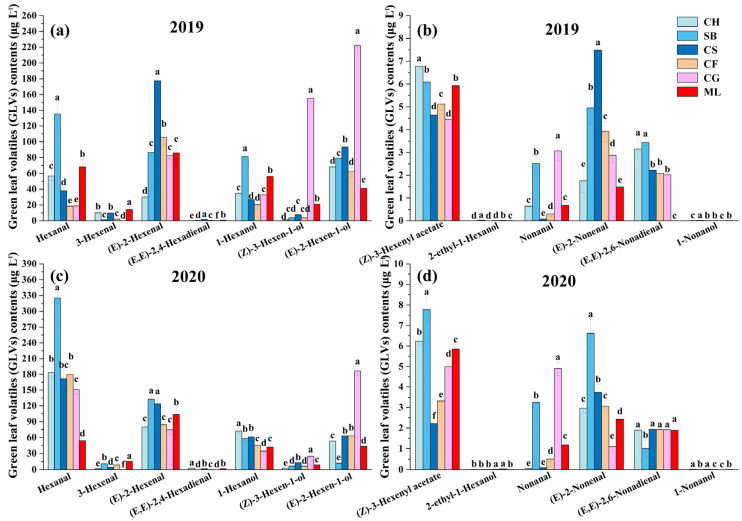
GLV concentrations in six grape varieties at harvest in the 2019 and 2020 vintages. (**a**) 2019 GLV compounds; (**b**) 2019 GLV compounds continue; (**c**) 2020 GLV compounds; (**d**) 2020 GLV compounds continue. Different letters show significant differences between treatments by Tukey’s test (*p* ≤ 0.05) (Consult the online version of this article for an elucidation of the color codes mentioned in the figure legend).

Brix is widely regarded as a reliable maturity indicator. Higher Brix levels typically signify greater ripeness, which is associated with not only increased sugar concentration but also alterations in the grape’s aromatic compound profile. Among the six grape varieties harvested, 13 GLVs were detected (Figure 2). The main GLVs in all mature 2019 berries were (E)-2-hexenal and (E)-2-hexen-1-ol. (E)-2-hexenal makes up 14.01–47.71% of the total GLVs composition, whereas (E)-2-hexen-1-ol accounts for 13.93–42.34% (Figure 2a,b). Hexanal and (E)-2-hexenal constituted the predominant components in mature grapes in 2020, with hexanal making up 31.26–57.51% of the total GLV contents and (E)-2-hexenal accounting for 15.42–27.73% (Figure 2c,d). This suggests that, despite the varietal differences in sugar accumulation, as reflected by Brix levels, certain characteristic compounds were universally present in grape varieties, contributing to the baseline aromatic complexity of wines.

Regarding C6 alcohols, at harvest, all grapes in both vintages contained 1-hexanol, (E)-2-hexen-1-ol, 2-ethyl-1-hexanol, and (Z)-3-hexen-1-ol. Cabernet Gernischt had the greatest C6 alcohol concentration in 2019 and 2020 among the six mature varieties, while Cabernet Franc had the lowest level in 2019 and Sauvignon Blanc in 2020. 1-hexanol enhances the perception of fruity, floral, and green notes in grapes [30]. In both vintages, Sauvignon Blanc and Cabernet Sauvignon exhibited higher 1-hexanol concentrations. (E)-2-hexen-1-ol imparts fruity aromas to grapes, produced through the catalytic action of ADH genes from (E)-2-hexenal [31]. According to the C6 alcohol profile, (E)-2-hexen-1-ol made up 14.16–74.43% among all grapes in both years. Cabernet Gernischt had the greatest concentrations of (E)-2-hexen-1-ol among all the types, with 222.21 μg L^−1^ in 2019 and 186.46 μg L^−1^ in 2020, similar to its overall C6 alcohol values. 

Four distinct C6 aldehydes were identified in ripe berries. (E)-2-hexenal and hexanal, the primary C6 aldehydes, were found in all grape types at harvest, in line with prior research on fresh grape varietals [5,32]. (E)-2-hexenal concentrations in mature berries varied between 30.05 and 177.74 μg L^−1^ over the two years. All the mature grapes had higher hexanal contents in 2020 than in 2019. In particular, mature Sauvignon Blanc had the greatest hexanal levels in 2019 and 2020. Consistent with our findings, (E)-2-hexenal and hexanal were the predominant C6 volatiles in the six wild grape species [9,25]. These compounds might impart a rich grassy and tomato flavor to grapes [33]. Wang et al. [11] also suggested that hexanal production is influenced by the growing environment and variety of grape plants.

The GLV profiles showed (Z)-3-hexenyl acetate as the only C6 ester. In 2019, the (Z)-3-hexenyl acetate content was highest in Chardonnay, and, in 2020, it was highest in Sauvignon Blanc. Some studies showed that (Z)-3-hexenyl had a minor impact on grape fragrance [23].

**Figure 3:**
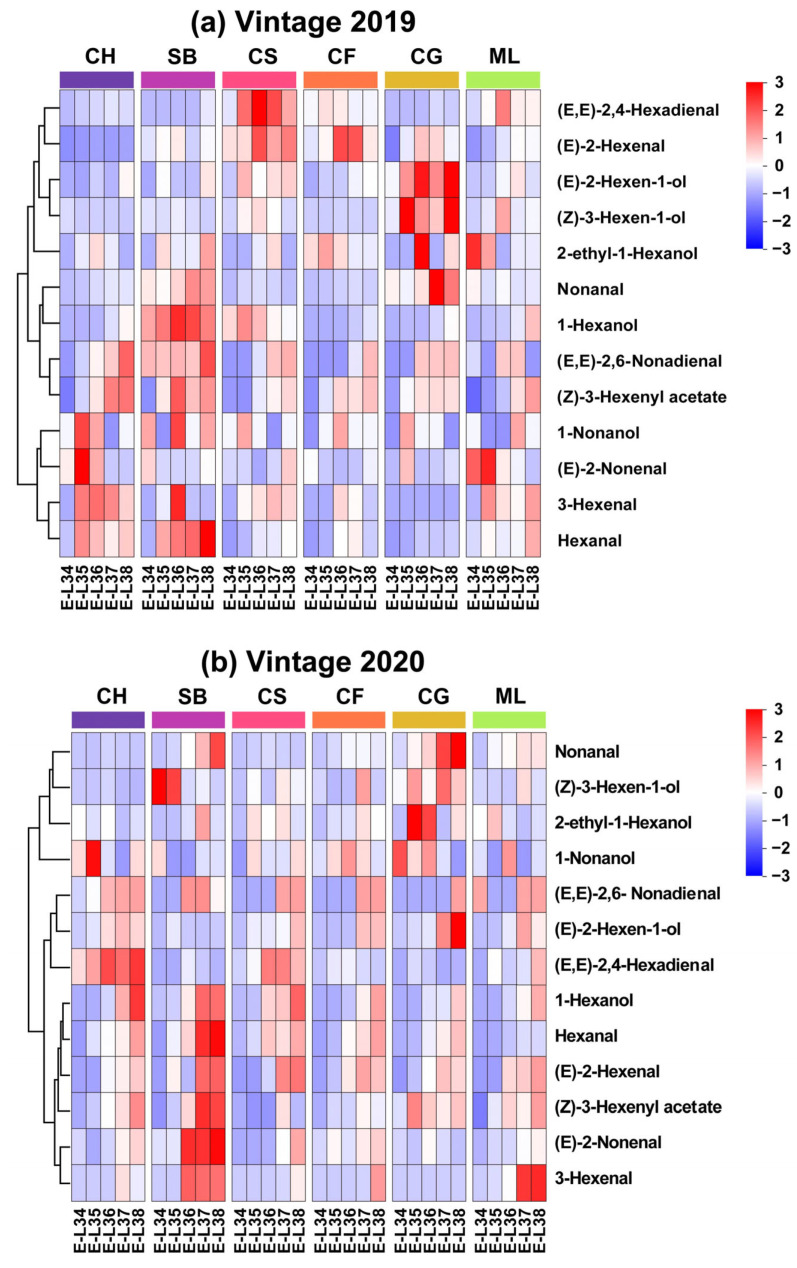
Heatmaps showing quantified GLVs in six grape types for the 2019 and 2020 vintages. (Consult the online version of this article for an elucidation of the color codes mentioned in the figure legend).

Four distinct C9 compounds were identified in ripe berries. Nonanal, (E)-2-nonenal, 1-nonanol, and (E, E)-2,6-nonadienal were detected in all grapes. Minimal quantities of 1-nonanol found in wine grapes (0.00–0.02 μg L^−1^) were consistent with a previous study [11]. In future studies, implementing comprehensive two-dimensional gas chromatography (2D GC) analysis could enhance detection accuracy and reduce detection thresholds for this set of chemicals [18,34]. The levels of (E)-2-nonenal and (E, E)-2,6-nonadienal were between 1.10–7.49 μg L^−1^ and 0–3.44 μg L^−1^, accordingly, beyond their smell thresholds of 0.08 and 0.02 μg L^−1^ [13,35]. The evolution of the GLV profiles in 2019 and 2020, respectively, are shown in Figure 3a,b. Our findings suggest that the development of the GLV profile varies not only among grape varieties but also among different vintages of the same variety suggested that this variation might be attributed to genetic factors and environmental influences. Below, we describe the variations in the GLV profiles of the six grape varietals as they progress through the stages of berry maturation.

The heatmap of concentrations reveals that GLV profiles are grouped into three distinct clusters. In 2019, 3-hexenal and hexanal exhibited a trend of increasing and then decreasing in Cabernet Franc and Cabernet Gernischt. A similar evolutionary pattern was observed in the remaining four grape varieties, with higher concentrations during the ripening period and lower concentrations at harvest (Figure 3a). 1-hexanol, (E, E)-2,6-nonadienal and (Z)-3-hexenyl acetate showed consistent patterns of rise in all six types, reaching their highest levels at the mature stage as they grew and developed. Some GLVs existed only at specific stages in typical varieties. For instance, the levels of (E)-2-hexenal and (E, E)-2,4-hexadienal are lower across the varieties at E-L 34. Regarding (E)-2-Hexen-1-ol and (Z)-3-Hexen-1-ol, Cabernet Gernischt exhibited the highest abundance during berry development, while Chardonnay had the lowest. In 2020, hexanal, 1-hexanol, (Z)-3-hexenyl acetate, and 3-hexenal across the six varieties matured into the same category, gradually increasing in content with growth and development, and peaking near maturity across the examined varieties (Figure 3b). Similar to the year 2019, the contents of 1-hexanol, hexanal and (Z)-3-hexenyl acetate increased with growth and development in 2020, peaking at the mature stage. However, (E)-2-nonenal and 3-hexenal had inconsistent evolution patterns over the two years.

The differences in the accumulation patterns of C6 and C9 aldehydes among different grape varieties highlight the complex interplay of genetic and environmental factors that influence grape aroma characteristics. As reported by Wang et al. [30], the total content of C6 aldehydes was greater in 2020, indicating a strong environmental impact, possibly related to climatic conditions. This observation is crucial for grape growers and winemakers, as it implies that annual climatic variations can significantly affect the aromatic compounds in grapes, thus influencing the quality of the wines. Furthermore, the variation in the peak accumulation stages of different grape varieties underscores the importance of determining the exact timing of grape harvesting to optimize the aroma profile. This knowledge can aid in developing targeted viticultural practices to enhance the aromatic characteristics of wines.

### 3.4. GLV Evolution Pattern Recognition

**Figure 4:**
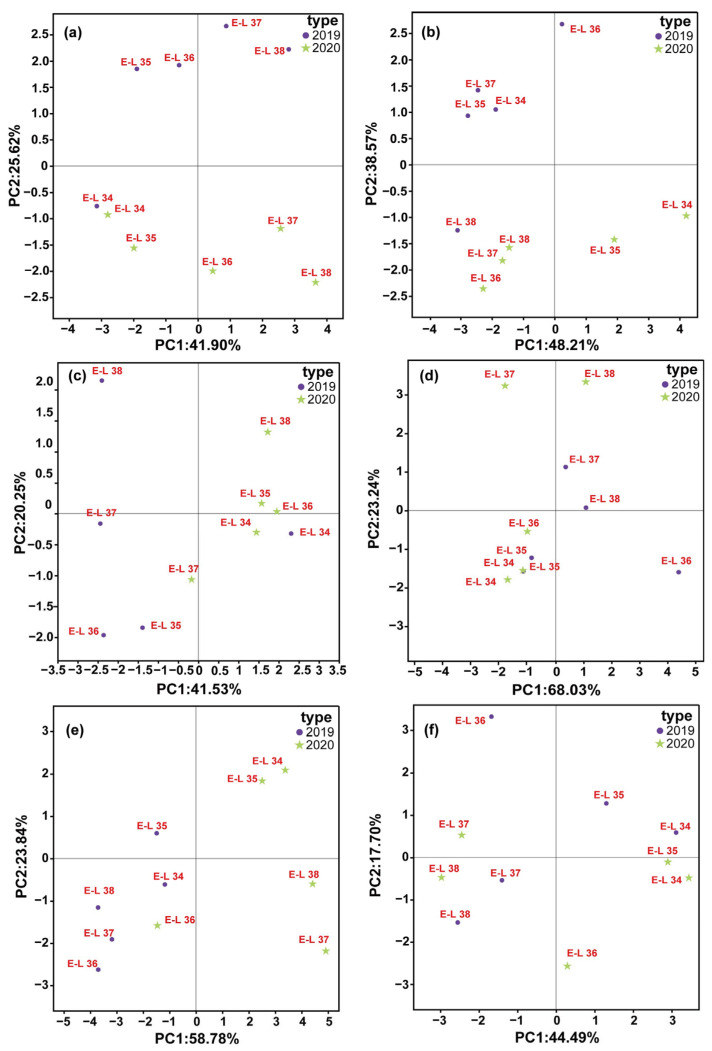
PCA Biplot showing the variation in GLVs generation during different phases of berry evolution in the 2019 (●) and 2020 (★) vintage. Legend: (**a**) Chardonnay, (**b**) Sauvignon Blanc, (**c**) Cabernet Sauvignon, (**d**) Cabernet Franc, (**e**) Cabernet Gernischt and (**f**) Marselan. (Consult the online version of this article for an elucidation of the color codes mentioned in the figure legend).

GLVs are mostly produced through the LOX-HPL pathway, and their production is influenced by grape farming and environmental conditions [6]. Genetic profile plays a major role in regulating GLV synthesis [36]. Prior research has shown that C6 volatile components differ among various fresh grape cultivars [37]. There is limited research on the development of GLV profiles in a diverse range of wine grapes in the Loess Plateau region. Therefore, principal component analysis (PCA) was applied to the GLV profiles of six wine grape varietals during a two-year period, as shown in Figure 4.

The synthesis of GLVs is controlled by environmental and viticultural conditions, as well as the genetic traits of the grape variety [9]. PCA was utilized to examine the variations in GLV components across different cultivars during fruit development (Figure 4a–f). The first two functions accounted for over 60.00% of the variance across all the types. The PCA results indicated that the GLV components of the six varieties exhibited noticeable differences at various growth stages. Furthermore, analysis of berry GLVs profiles showed significant differences between the E-L34 and E-L38 period across the six varieties. Previous studies on fresh cultivation varieties also indicated that grapes have distinctive C6 volatile contents at harvest, which aligns with our findings [38].

Building upon the PCA findings, we subsequently performed a Partial Least Squares Discriminant Analysis (PLS-DA) to more effectively discern the variations in GLV profiles during the maturation process of grape berries across two vintages, 2019 and 2020 (Figure S1a,b). In both vintages, the first principal component (PC1) and the second principal component (PC2) represent substantial proportions of the variation, with PC1 accounting for 20.4% in 2019 and 33.2% in 2020, while PC2 captures 8.6% and 15.7%, respectively. The score plots illustrate a distinct segregation of samples along PC1, indicating a pronounced variance in GLV profiles as a function of the maturation stage.

To further investigate the impact of specific GLVs on the separation observed in the PLS-DA, Variable Importance in Projection (VIP) scores derived from a PLS-DA were utilized (Figure S1c,d). Compounds with VIP scores greater than 1 are considered significant contributors to the model. In 2019, 1-hexanol, hexanal, (E, E)-2,6-nonadienal, and 3-hexenal were identified as significant due to their VIP scores surpassing this threshold. Similarly, in 2020, 3-hexenal, (E)-2-nonenal, 1-nonanol, and (E)-2-hexen-1-ol were distinguished for their substantial contributions, as reflected by the VIP scores above. Notably, 3-hexenal exhibited high VIP scores in both years, highlighting their influence on the grape variety classification. Such analysis is crucial for identifying biochemical markers impacting grape quality and wine flavor.

### 3.5. Aroma Activity Analysis

Odor activity value (OAV) was calculated by assessing the ratio of the compound concentration to its odor threshold in water [8]. Components with an OAV of more than 1 made a substantial contribution to the grape scent, as detailed in Tables S3–S8. Among all the mature berries from 2019 and 2020, there were nearly 7 and 8 GLV compounds, respectively, with an OAV > 1. Out of the six fully developed grape types assessed in both years, three odor-active GLVs with OAV exceeding 30 were identified as 3-hexenal, (E)-2-nonenal, and (E, E)-2,6-nonadienal, which are likely to play a substantial role in grape scent.

In 2019, Chardonnay at E-L 35 had the highest (E)-2-nonenal OAV, followed by Marselan. Sauvignon Blanc had a greater (E, E)-2,6-nonadienal OAV at harvest, with mature Chardonnay and Cabernet Sauvignon berries having (E, E)-2,6-nonadienal OAVs of 157.50 and 111.12, respectively. The compound (E, E)-2,6-nonadienal had the highest OAV among all the mature berries in 2020, followed by (E)-2-nonenal and 3-hexenal. Mature Cabernet Sauvignon had the highest (E, E)-2,6-nonadienal OAV, with mature Sauvignon Blanc having higher (E)-2-nonenal and hexanal OAVs, which likely had significant impact on the creation of aroma.

Descriptors are commonly employed to describe volatile substances in odor compounds. Aroma compound descriptors are classified into six kinds: sweet, fruity, green, citrus, chemical, and floral [39]. The aroma profiles of the six grape types differed, with mature Sauvignon Blanc berries exhibiting the most pronounced green aroma due to elevated levels of hexanal. The odor Cabernet Sauvignon, Cabernet Gernischt, and Cabernet Franc peaked at maturity. The aroma profile of the berries changed as they matured, affected by the changing seasons. In 2020, green notes were more pronounced than other odors in all six grape varieties. The aroma characteristics of the six grape varieties generally increased gradually with growth and development. During harvest, Sauvignon Blanc grapes exhibited the strongest green aroma.

This research contributes to a deeper understanding of the chemical basis of grape aroma and flavor, which is crucial in the field of oenology. Furthermore, investigating the effects of these compounds on the final product quality will be valuable. Although our study provides significant insights, it is limited to a few grape varieties. Extending this research to a broader range of varieties could provide a more comprehensive understanding of the variability in aroma compounds.

### 3.6. Expression of Genes in LOX-HPL Pathway

**Figure 5:**
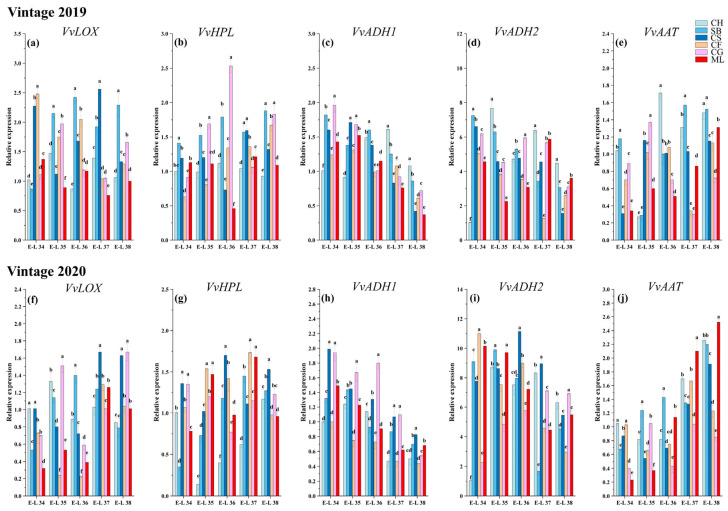
Gene expression levels of LOX-HPL pathway genes in six grape types. The data were normalized against the VviActin expression data. (**a**) 2019 *VvLOX*; (**b**) 2019 *VvHPL*; (**c**) 2019 *VvADH1*; (**d**) 2019 *VvADH2*; (**e**) 2019 *VvAAT*; (**f**) 2020 *VvLOX*; (**g**) 2020 *VvHPL*; (**h**) 2020 *VvADH1*; (**i**) 2020 *VvADH2*; (**j**) 2020 *VvAAT.* Different letters show significant differences between treatments by Tukey’s test (*p* ≤ 0.05) (Consult the online version of this article for an elucidation of the color codes mentioned in the figure legend).

Considering the discernible variation in the concentrations of most GLV compounds across different sampling times and grape varieties, coupled with the fact that C6/C9 volatile compounds are directly synthesized via the LOX-HPL pathway, our study concentrated on analyzing genes that encode the enzymes functional in the LOX, HPL, ADH, and AAT within this pathway. This analysis was performed using RT-qPCR. The levels of gene expression related to the accumulation of GLVs are shown in Figure 5.

**Figure 6:**
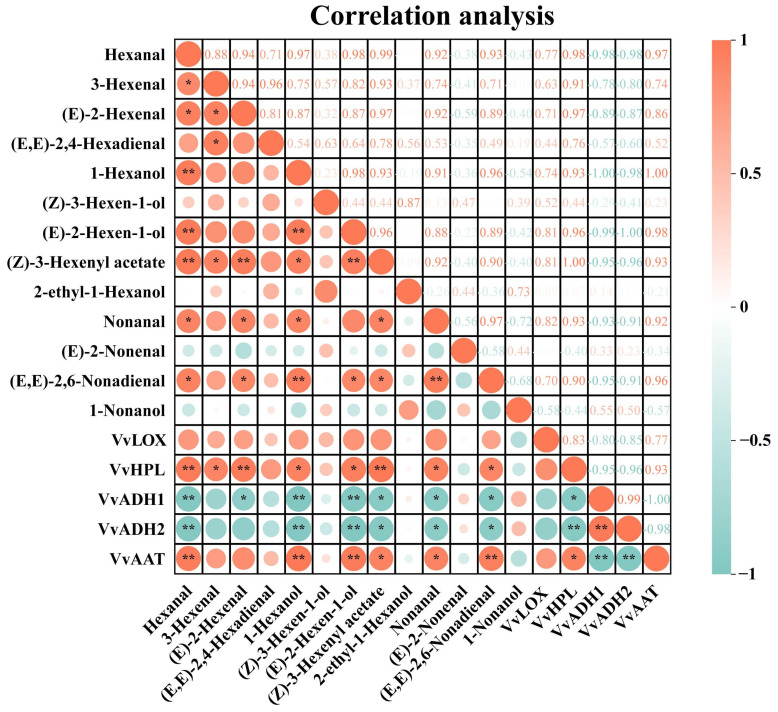
Correlation analysis was conducted on the quantities of GLVs and the level of LOX-HPL pathway genes in six grape types in this study. The color scale in this study represents correlations from −1 to 1, with the orange color denoting a positive association between GLVs concentrations and related genes, and green indicating a negative association. Cells are colored based on statistical value and marked with asterisks. * *p*-value < 0.05; ** *p*-value < 0.01. (Consult the online version of this article for an elucidation of the color codes mentioned in the figure legend).

Lipoxygenase (LOX) catalyzes the synthesis of hydroperoxides, which are direct precursors for aldehyde synthesis [16]. The expression patterns of the *VvLOX* genes varied among the six types during development (Figure 5a,f). In this study, the activity of the *VvLOX* gene exhibited fluctuating trends throughout the growth and development stages of six grape varieties in 2019, while showing a notably upward trend overall in 2020. This indicates that the varieties’ responses to environmental or developmental factors may vary between years, suggesting a nuanced interaction between genetic expression and external conditions. The study found that the activity of the *VvLOX* gene gradually increased during the growth and development of the six grape varieties, especially in 2020. In 2019, the highest expression of the *VvLOX* genes was detected in Cabernet Franc and Cabernet Sauvignon during E-L 34. In Sauvignon Blanc, Cabernet Sauvignon, and Cabernet Gernischt, the expression peaked during the ripening stages. This discovery implied that a cultivar-specific temporal expression pattern could influence the timing and intensity of aroma compound synthesis. In 2020, the highest expression of the *VvLOX* gene was detected in Cabernet Sauvignon and Chardonnay during E-L 34. In the early maturation stages, E-L 34, 35, and 36, the expression of *VvLOX* in Cabernet Franc and Marselan was significantly lower than that in the other varieties. During the ripening stages, the *VvLOX* gene exhibited markedly elevated expression in Cabernet Sauvignon and Cabernet Gernischt relative to other grape varieties. Despite this, the levels of C6/C9 aldehydes in these two varieties did not significantly surpass those in other grapes at the same stages. This observation indicates a potential delay in the *VvLOX*-mediated regulation of C6/C9 aldehyde accumulation in Cabernet Sauvignon and Cabernet Gernischt (Figure 2 and Figure 6).

Hydroperoxide lyase (HPL) breaks down fatty acid hydroperoxides into C6/C9 aldehydes [6,40]. Over the two years, the levels of *VvHPL* in Chardonnay and Sauvignon Blanc generally increased with growth and development, while those in the other varieties generally increased initially and then decreased during the maturation period, suggesting genetic differences in the regulation of the LOX-HPL pathway (Figure 5b,g). In 2019, during E-L 34, the highest expression of *VvHPL* occurred in Sauvignon Blanc, followed by Cabernet Sauvignon and Marselan, which could impact the early formation of aroma compounds. In the early maturation stages E-L 35 and 36, the levels of *VvHPL* in Cabernet Gernischt were markedly greater than those in other varieties. At E-L 38, the expression in Sauvignon Blanc and Cabernet Gernischt was notably higher compared to other varietals. This is consistent with the development of aldehydes (Figure 2). In 2020, during the E-L 34 stage, the expression of *VvHPL* in Cabernet Sauvignon and Cabernet France was significantly greater than that in the other varieties. At the mature stage, the expression in Cabernet Sauvignon was significantly greater than that in the other varieties. Contrasting this, the 2020 vintage revealed a marked deviation in this trend within the Chardonnay variety, where gene expression levels first decreased dramatically before showing a notable increase. The initial decrease in gene expression could be attributed to the rapid rise in temperatures and the subsequent stress conditions experienced by the vines. As the season progressed, an increase in humidity and rainfall, alongside stable sunshine hours, may have created conditions conducive to the recovery and enhancement of *VvHPL* gene activity, particularly at the later stages of grape development. Chardonnay and Sauvignon Blanc show a positive correlation with (E, E)-2,4-hexadienal and hexanal, in contrast to Marselan, which shows a negative correlation with hexanal. This pattern suggests the presence of distinct *VvHPL* regulatory mechanisms in different grape varieties. 

Alcohol dehydrogenase (ADH) catalyzes the synthesis of alcohols [31]. The transcript levels of *VvADH1* and *VvADH2* showed an overall declining trend in the six varieties over the two years. Qian et al. [23] found that the levels of *VvADH* in Muscat Tchervine, Gewurztraminer and Syrah decreased after the veraison stage, similar to the results of our studies. In 2019, the *VvADH1* gene exhibited the highest expression in Chardonnay at harvest, while Marselan having the lowest expression throughout the developmental process (Figure 5c). In 2020, the expression of the *VvADH1* gene was greatest in Chardonnay at harvest, while Cabernet Franc exhibited relatively low *VvADH1* gene expression throughout the developmental process (Figure 5h). *VvADH2* is the most abundant among all the *VvADHs*. In 2019, at harvest, Chardonnay exhibited the highest *VvADH2* expression (Figure 5d). In 2020, Chardonnay, Sauvignon Blanc and Cabernet Sauvignon exhibited a trend of increasing and then decreasing expression levels during the growth and development process. Cabernet Gernischt exhibited an increase in *VvADH2* expression, while the other varieties generally exhibited a gradual decrease in *VvADH2* expression with growth and development (Figure 5i). Among them, Cabernet Sauvignon exhibited the highest expression, and Cabernet Franc exhibited the lowest.

Alcohol acyltransferase (AAT) catalyzes the conversion of low-molecular-weight alcohols into esters [11]. Over two years, *VvAAT* gene expression in Cabernet Gernischt decreased with maturity, while, in other varieties, it generally increased with growth. In 2019, Chardonnay exhibited the highest *VvAAT* gene expression at E-L 36 phases (Figure 5e). The expression of the *VvAAT* gene in Sauvignon Blanc was significantly greater than that in the other varieties at mature stage. *VvAAT* gene expression in the later stages of maturation in 2020 was significantly greater than that in 2019, possibly due to the lower rainfall in 2020 (Figure 5j). At the mature stage, the expression of the *VvAAT* gene in Mareslan was significantly greater than that in the other varieties. This finding suggested that a cultivar-specific temporal expression pattern could influence the timing and intensity of aroma compound synthesis. In the berries of Chardonnay and Sauvignon Blanc, a pronounced transcription of *VvAAT* correlates with elevated levels of C6 esters. This indicates a significant positive relationship between *VvAAT* expression and C6 ester accumulation in these grape varieties.

### 3.7. Correlation Analysis

To improve our understanding of these results, we conducted a correlation analysis between the expression of C6/C9 aromatic substances and LOX-HPL pathway genes in grapes, as shown in Figure 6. Among the six wine grape varieties, *VvLOX* exhibited a positive correlation with most C6/C9 volatile compounds, although the correlation was not significant. *VvHPL* exhibited a highly significant positive correlation with hexanal, (Z)-3-hexenyl acetate, and (E)-2-hexenal. It was also significantly positively correlated with 3-hexenal, (E)-2-hexen-1-ol, 1-hexanol, nonanal, and (E, E)-2,6-nonadienal. Both *VvADH1* and *VvADH2* were strongly negatively correlated with hexanal, (E)-2-hexen-1-ol, and 1-hexanol. These genes were significantly negatively correlated with (E, E)-2,6-nonadienal, (Z)-3-hexenyl acetate, and nonanal. *VvAAT* had a highly significant positive correlation with 1-hexanol, hexanal, (E, E)-2,6-nonadienal and a significant positive correlation with (Z)-3-hexenyl acetate and nonanal. This discovery suggests a strong connection between the activity of these LOX-HPL pathway genes and the build-up of C6 volatiles. Additionally, hexanal was significantly positively correlated with 3-hexenal, 1-hexanol, and (E, E)-2,6-nonadienal.

Recent studies have demonstrated that viticultural practices and varietal differences regulate the transcription of genes in the LOX-HPL pathway [41,42]. Specifically, gene transcription levels within the LOX-HPL pathway vary among wine grape varieties and are closely linked to the production of C6/C9 volatile compounds. Notably, Syrah grapes exhibit a higher concentration of C6 aldehydes, suggesting that increased expression of *VvLOX* and *VvHPL* genes contributes to this heightened accumulation [43]. Ju et al. [9] further highlights the variability in C6 compound concentrations across six varieties of prickly grapes, a variation that correlates with the expression levels of the *VdLOX* and *VdHPL* genes. 

Our findings further reveal that (E)-2-hexenal, hexanal, and (E)-2-hexen-1-ol are most abundant in six grape varieties (Figure 3). There is a particularly strong positive correlation between the levels of hexanal and (E)-2-hexenal and the expression of *VvHPL*. These findings point to *VvHPL* as a potential key gene influencing varietal differences in GLV profiles.

## 4. Conclusions

This research assessed how GLV profiles vary with grape variety during the ripening of six wine grape types in the Loess Plateau region. A total of 13 GLVs were profiled: 4 kinds of C6 aldehydes, 4 kinds of C6 alcohols, 1 kind of C6 ester, 3 kinds of C9 aldehydes, and 1 kind of C9 alcohol. The predominant components detected in the mature berries’ GLV profiles in 2019 were (E)-2-hexenal and (E)-2-hexen-1-ol, whereas, in 2020, hexanal and (E)-2-hexenal were the main components. Changes in the evolution patterns of total GLV contents were influenced by grape variety and season. With the exception of the 2019 Cabernet Franc variety, the overall GLV content of the six types generally increased during growth and development, reaching a peak or stabilizing at harvest. Sauvignon Blanc, Cabernet Gernischt, and Cabernet Sauvignon had the highest total levels compared to other varietals. Over the two years, the evolution patterns of 1-hexanol, hexanal and (Z)-3-hexenyl acetate in the six grape varieties were similar, with the contents of these compounds gradually increasing with growth and development and peaking at maturity. PLS-DA analysis revealed 3-hexenal’s high VIP scores across two years, underscoring its critical role in grape variety classification. We also assessed the gene expression of LOX-HPL pathway-related genes at various phenological stages in the six grape types. In the Loess Plateau region, the six wine grape varieties were found to have the highest levels of (E)-2-hexenal, hexanal, and (E)-2-hexen-1-ol, with hexanal and (E)-2-hexenal accumulation showing a highly significant positive correlation with the expression of *VvHPL*. These results suggest that *VvHPL* could be a candidate gene responsible for the varietal differences in GLV compounds observed in the Loess Plateau area, indicating its pivotal role in the biosynthesis and accumulation of these key aroma compounds in grapes. The results of this study provide insights into the GLV profiles and related genes of various wine grape varieties at different stages. Meanwhile, the present results could assist winemakers in improving the “green” aroma in wine production by choosing the optimal grape varietal and maturity to create a good wine aroma balance.

## Data Availability

The original contributions presented in the study are included in the article/Supplementary Materials, further inquiries can be directed to the corresponding authors.

## Supplementary Materials

The following supporting information can be downloaded at: https://www.mdpi.com/article/10.3390/foods13081207/s1, Table S1: Temperature and rainfall conditions during vintages 2019 and 2020; Table S2: Physicochemical parameters in the berries of six *Vitis vinifera* L. cultivars at harvest; Table S3: Odour thresholds (OT), odour description, Odour activity values (OAVs) of green leaf volatiles (GLVs) in Chardonnay at different stages of the berry maturation in vintage 2019 and vintage 2020; Table S4: Odour thresholds (OT), odour description, Odour activity values (OAVs) of green leaf volatiles (GLVs) in Sauvignon Blanc at different stages of the berry maturation in vintage 2019 and vintage 2020; Table S5: Odour thresholds (OT), odour description, Odour activity values (OAVs) of green leaf volatiles (GLVs) in Cabernet Sauvignon at different stages of the berry maturation in vintage 2019 and vintage 2020; Table S6: Odour thresholds (OT), odour description, Odour activity values (OAVs) of green leaf volatiles (GLVs) in Cabernet Franc at different stages of the berry maturation in vintage 2019 and vintage 2020; Table S7: Odour thresholds (OT), odour description, Odour activity values (OAVs) of green leaf volatiles (GLVs) in Cabernet Gernischt at different stages of the berry maturation in vintage 2019 and vintage 2020; Table S8: Odour thresholds (OT), odour description, Odour activity values (OAVs) of green leaf volatiles (GLVs) in Maselan at different stages of the berry maturation in vintage 2019 and vintage 2020; Table S9: Quantitative ion, quantitative standards and calibration curves for quantification of volatile compounds; Table S10: Primer used in real-time PCR; Figure S1: Partial Least Squares Discriminant Analysis biplot illustrating the pattern of green leaf volatiles (GLVs) at different stages of the berry maturation in vintage 2019 (a) and vintage 2020 (b). VIP diagrams illustrate key differential compounds in vintage 2019 (c) and vintage 2020 (d). Legend: ▲-Chardonnay, ●-Sauvignon Blanc, ◆-Cabernet Sauvignon, ■-Cabernet Franc, ▲-Cabernet Gernischt, ●-Marselan.

## References

[1] Li C., Chen H., Li Y., Du T., Jia J., Xi Z. (2022). The Expression of Aroma Components and Related Genes in Merlot and Marselan Scion-Rootstock Grape and Wine. Foods.

[2] He Y., Wang X., Li P., Lv Y., Nan H., Wen L., Wang Z. (2023). Research progress of wine aroma components: A critical review. Food Chem..

[3] Yue X., Ju Y., Zhang H., Wang Z., Xu H., Zhang Z. (2022). Integrated transcriptomic and metabolomic analysis reveals the changes in monoterpene compounds during the development of Muscat Hamburg (*Vitis vinifera* L.) grape berries. Food Res. Int..

[4] Godshaw J., Hjelmeland A.K., Zweigenbaum J., Ebeler S.E. (2019). Changes in glycosylation patterns of monoterpenes during grape berry maturation in six cultivars of *Vitis vinifera*. Food Chem..

[5] Chen K., Wen J., Ma L., Wen H., Li J. (2019). Dynamic changes in norisoprenoids and phenylalanine-derived volatiles in off-vine *Vidal blanc* grape during late harvest. Food Chem..

[6] Xu X.Q., Cheng G., Duan L.L., Jiang R., Pan Q.H., Duan C.Q., Wang J. (2015). Effect of training systems on fatty acids and their derived volatiles in Cabernet Sauvignon grapes and wines of the north foot of Mt. Tianshan. Food Chem..

[7] Xi X., Zha Q., He Y., Tian Y., Jiang A. (2020). Influence of cluster thinning and girdling on aroma composition in ‘Jumeigui’ table grape. Sci. Rep..

[8] Jaillon O., Aury J.-M., Noel B., Policriti A., Clepet C., Casagrande A., Choisne N., Aubourg S., Vitulo N., Jubin C. (2007). The grapevine genome sequence suggests ancestral hexaploidization in major angiosperm phyla. Nature.

[9] Ju Y.L., Yue X.F., Cao X.Y., Wei X.F., Fang Y.L. (2021). First study on the fatty acids and their derived volatile profiles from six Chinese wild spine grape clones (*Vitis davidii* Foex). Sci. Hortic..

[10] Moreno D., Valdes E., Uriarte D., Gamero E., Talaverano I., Vilanova M. (2017). Early leaf removal applied in warm climatic conditions: Impact on Tempranillo wine volatiles. Food Res. Int..

[11] Wang P., Yu A., Ji X., Mu Q., Haider M.S., Wei R., Leng X., Fang J. (2022). Transcriptome and metabolite integrated analysis reveals that exogenous ethylene controls berry ripening processes in grapevine. Food Res. Int..

[12] Yue X., Ju Y., Zhang T., Yu R., Xu H., Zhang Z. (2023). Application of salicylic acid to cv. Muscat Hamburg grapes for quality improvement: Effects on typical volatile aroma compounds and anthocyanin composition of grapes and wines. LWT-Food Sci. Technol..

[13] Wang J., Abbey T., Kozak B., Madilao L.L., Tindjau R., Del Nin J., Castellarin S.D. (2019). Evolution over the growing season of volatile organic compounds in Viognier (*Vitis vinifera* L.) grapes under three irrigation regimes. Food Res. Int..

[14] Cheng X., Liang Y., Zhang A., Wang P., He S., Zhang K., Wang J., Fang Y., Sun X. (2021). Using foliar nitrogen application during veraison to improve the flavor components of grape and wine. J. Sci. Food Agric..

[15] Xie S., Wu G., Ren R., Xie R., Yin H., Chen H., Yang B., Zhang Z., Ge M. (2023). Transcriptomic and metabolic analyses reveal differences in monoterpene profiles and the underlying molecular mechanisms in six grape varieties with different flavors. Lwt-Food Sci. Technol..

[16] Yao H., Jin X., Feng M., Xu G., Zhang P., Fang Y., Xu T., Meng J. (2021). Evolution of volatile profile and aroma potential of table grape Hutai-8 during berry ripening. Food Res. Int..

[17] Alem H., Rigou P., Schneider R., Ojeda H., Torregrosa L. (2019). Impact of agronomic practices on grape aroma composition: A review. J. Sci. Food Agric..

[18] Luo J., Brotchie J., Pang M., Marriott P.J., Howell K., Zhang P. (2019). Free terpene evolution during the berry maturation of five *Vitis vinifera* L. cultivars. Food Chem..

[19] Antalick G., Suklje K., Blackman J.W., Meeks C., Deloire A., Schmidtke L.M. (2015). Influence of Grape Composition on Red Wine Ester Profile: Comparison between Cabernet Sauvignon and Shiraz Cultivars from Australian Warm Climate. J. Agric. Food Chem..

[20] Tang K., Xi Y.R., Ma Y., Zhang H.N., Xu Y. (2019). Chemical and Sensory Characterization of Cabernet Sauvignon Wines from the Chinese Loess Plateau Region. Molecules.

[21] Coombe B.G. (1995). Growth Stages of the Grapevine: Adoption of a system for identifying grapevine growth stages. Aust. J. Grape Wine Res..

[22] Wen Y.Q., He F., Zhu B.Q., Lan Y.B., Pan Q.H., Li C.Y., Reeves M.J., Wang J. (2014). Free and glycosidically bound aroma compounds in cherry (*Prunus avium* L.). Food Chem..

[23] Qian X., Liu Y., Zhang G., Yan A., Wang H., Wang X., Pan Q., Xu H., Sun L., Zhu B. (2019). Alcohol acyltransferase gene and ester precursors differentiate composition of volatile esters in three interspecific hybrids of *Vitis labrusca xV. Vinifera* during berry development period. Food Chem..

[24] Zhou X., Liu S., Gao W., Hu B., Zhu B., Sun L. (2022). Monoterpenoids Evolution and MEP Pathway Gene Expression Profiles in Seven Table Grape Varieties. Plants.

[25] Lin J., Massonnet M., Cantu D. (2019). The genetic basis of grape and wine aroma. Hortic. Res..

[26] Lu H.C., Chen W.K., Wang Y., Bai X.-J., Cheng G., Duan C.Q., Wang J., He F. (2022). Effect of the Seasonal Climatic Variations on the Accumulation of Fruit Volatiles in Four Grape Varieties Under the Double Cropping System. Front. Plant Sci..

[27] Geng K., Li D., Zhang J., Zhang Y., Zhan Z., Wang Z. (2022). Evolution of volatile aroma compounds and amino acids in Cabernet Gernischt grape berries (*Vitis vinifera* L.): Comparison of different training systems for mechanical soil burial. Foods.

[28] Tian M.B., Ma W.H., Xia N.Y., Peng J., Hu R.Q., Duan C.Q., He F. (2023). Soil variables and reflected light revealed the plasticity of grape and wine composition: Regulation of the flavoromics under inner row gravel covering. Food Chem..

[29] Zhang Z., Qiao D., He L., Pan Q., Wang S. (2022). Effects of vine top shading on the accumulation of C6/C9 compounds in’Cabernet Sauvignon’ (*Vitis vinifera* L.) grape berries in northwestern China. J. Sci. Food Agric..

[30] Wang H., Wang X., Yan A., Liu Z., Ren J., Xu H., Sun L. (2023). Metabolomic and transcriptomic integrated analysis revealed the decrease of monoterpenes accumulation in table grapes during long time low temperature storage. Food Res. Int..

[31] Khakimov B., Bakhytkyzy I., Fauhl-Hassek C., Engelsen S.B. (2022). Non-volatile molecular composition and discrimination of single grape white of chardonnay, riesling, sauvignon blanc and silvaner using untargeted GC-MS analysis. Food Chem..

[32] Choi K.O., Lee D.H., Park S.J., Im D., Hur Y.Y., Kim S.J. (2020). Changes in Biochemical and Volatile Flavor Compounds of Shine Muscat at Different Ripening Stages. Appl. Sci..

[33] Gonzalez-Barreiro C., Rial-Otero R., Cancho-Grande B., Simal-Gandara J. (2015). Wine Aroma Compounds in Grapes: A Critical Review. Crit. Rev. Food Sci. Nutr..

[34] Lukić I., Carlin S., Vrhovsek U. (2020). Comprehensive 2D gas chromatography with TOF-MS detection confirms the matchless discriminatory power of monoterpenes and provides in-depth volatile profile information for highly efficient white wine varietal differentiation. Foods.

[35] Aubert C., Chalot G. (2018). Chemical composition, bioactive compounds, and volatiles of six table grape varieties (*Vitis vinifera* L.). Food Chem..

[36] Wang Y., He Y.N., He L., He F., Chen W., Duan C.Q., Wang J. (2019). Changes in global aroma profiles of Cabernet Sauvignon in response to cluster thinning. Food Res. Int..

[37] Wu Y., Zhang W., Song S., Xu W., Zhang C., Ma C., Wang L., Wang S. (2020). Evolution of volatile compounds during the development of Muscat grape ‘Shine Muscat’ (*Vitis labrusca × V. vinifera*). Food Chem..

[38] Qian X., Sun L., Xu X.Q., Zhu B.Q., Xu H.Y. (2017). Differential Expression of *VvLOXA* Diversifies C6 Volatile Profiles in Some *Vitis vinifera* Table Grape Cultivars. Int. J. Mol. Sci..

[39] Abbas F., Ke Y., Yu R., Yue Y., Amanullah S., Jahangir M.M., Fan Y. (2017). Volatile terpenoids: Multiple functions, biosynthesis, modulation and manipulation by genetic engineering. Planta.

[40] Zhu B.Q., Xu X.Q., Wu Y.W., Duan C.Q., Pan Q.H. (2012). Isolation and characterization of two hydroperoxide lyase genes from grape berries. Mol. Biol. Rep..

[41] Souleyre E.J.F., Chagne D., Chen X., Tomes S., Turner R.M., Wang M.Y., Maddumage R., Hunt M.B., Winz R.A., Wiedow C. (2014). The AAT1 locus is critical for the biosynthesis of esters contributing to ‘ripe apple’ flavour in ‘Royal Gala’ and ‘Granny Smith’ apples. Plant J..

[42] Tesniere C., Davies C., Sreekantan L., Bogs J., Thomas M., Torregrosa L. (2006). Analysis of the transcript levels of *VvAdh1*, *VvAdh2* and *VvGrip4*, three genes highly expressed during *Vitis vinifera* L. berry development. Vitis.

[43] Qian X., Xu X.Q., Yu K.J., Zhu B.Q., Lan Y.B., Duan C.Q., Pan Q.H. (2016). Varietal Dependence of GLVs Accumulation and LOX-HPL Pathway Gene Expression in Four *Vitis vinifera* Wine Grapes. Int. J. Mol. Sci..

