# Supplementary material for: Comparative Analysis of the Evolution of Green Leaf Volatiles and Aroma in Six Vitis vinifera L. Cultivars during Berry Maturation in the Chinese Loess Plateau Region
Source: Foods. 2024 Apr 16;13(8):1207. doi: 10.3390/foods13081207 (PMC11049030; doi:10.3390/foods13081207)
Supplement: Supplementary file 1 [file foods-13-01207-s001.zip › foods-2938220-supplementary.pdf]

Table S1. Temperature and rainfall conditions during vintages 2019 and 2020.

	April	May	June	July	August	September
Vintage 2019						
Mean maximum temperature (°C)	23.20	28.60	33.50	35.00	31.40	27.70
Mean minimum temperature (°C)	10.99	15.62	21.62	23.73	21.99	17.14
Mean temperature (°C)	16.35	21.88	27.08	28.85	26.16	21.55
Mean relative humidity	49.50	32.40	37.80	44.50	54.10	52.40
Sum rainfall (mm)	40.60	3.20	75.20	35.00	66.30	111.90
Mean sunshine hours (h)	8.53	8.81	9.07	9.68	9.72	9.12
Vintage 2020						
Mean maximum temperature (°C)	22.74	29.66	32.26	31.92	30.45	27.42
Mean minimum temperature (°C)	8.55	15.99	20.75	21.88	22.10	16.80
Mean temperature (°C)	15.37	22.61	26.01	26.38	25.48	21.52
Mean relative humidity	39.04	40.64	47.68	58.20	68.39	56.38
Sum rainfall (mm)	30.80	62.70	55.40	77.70	183.70	10.80
Mean sunshine hours (h)	10.39	10.24	10.27	10.57	11.07	10.13

Table S2. Physicochemical parameters in the berries of six Vitis vinifera L. cultivars at harvest.

Vintages	Varieties	TSS (Brix)[a]	TA (g L^{-1})[b]	pH
2019	Chardonnay	19.29±0.11 d	5.84±0.05 a	4.16±0.01 c
	Sauvignon Blanc	19.94±0.36 c	5.47±0.14 b	4.29±0.02 a
	Cabernet Sauvignon	22.90±0.10 b	5.23±0.02 c	4.30±0.00 a
	Cabernet Franc	22.71±0.22 b	4.57±0.10 e	4.31±0.01 a
	Cabernet Gernischt	19.22±0.09 d	4.94±0.03 d	4.24±0.01 b
	Marselan	23.65±0.42 a	5.16±0.05 c	4.14±0.01 c
2020	Chardonnay	18.93±0.51 d	5.74±0.30 b	3.84±0.01 c
	Sauvignon Blanc	18.87±0.32 d	6.30±0.09 a	3.70±0.02 d
	Cabernet Sauvignon	21.07±0.60 b	4.02±0.04 d	4.01±0.00 b
	Cabernet Franc	22.53±0.29 a	5.61±0.34 b	4.15±0.04 a
	Cabernet Gernischt	19.97±0.06 c	4.92±0.17 c	4.20±0.03 a
	Marselan	22.47±0.25 a	5.64±0.21 b	3.97±0.03 b

Table S3. Odour thresholds (OT), odour description, Odour activity values (OAVs) of green leaf volatiles (GLVs) in Chardonnay at different stages of the berry maturation in vintage 2019 and vintage 2020.

Compounds	OT[a] (ppb in water)	Odour description	Aromatic series	2019 Chardonnay OAV[b]					2020 Chardonnay OAV[b]				
				E-L 34	E-L35	E-L36	E-L 37	E-L 38	E-L 34	E-L 35	E-L 36	E-L 37	E-L38
hexanal	4.5	Green, grassy, fruity	Green, fruity	3.34	17.79	13.87	9.84	12.65	0.00	14.09	20.03	24.09	40.77
3-hexenal	0.21	Green, grassy	Green	0.00	82.30	86.75	76.46	48.76	0.00	0.00	0.00	23.66	9.01
(E)-2-hexenal	17	Green, grassy	Green	0.99	1.39	1.71	1.56	1.77	0.48	0.45	2.98	3.73	4.71
(E,E)-2,4-hexadienal	10	Green, sweet, fruity	Green, fruity	0.00	0.00	0.00	0.00	0.00	0.01	0.01	0.02	0.02	0.02
1-hexanol	150	Green, fruity, flowery, grassy	Green, fruity	0.08	0.11	0.21	1.38	2.69	0.00	0.04	0.79	3.23	5.59
(Z)-3-hexen-1-ol	13	Green,lettuce-like, grassy	Green	0.08	0.02	0.04	0.02	0.01	0.06	0.05	0.08	0.03	0.03
(E)-2-hexen-1-ol	100	Fruity,ethereal	Fruity	1.19	0.61	3.22	1.70	6.85	1.28	2.30	4.99	6.46	5.30
(Z)-3-hexenyl acetate	8	Green; banana-like	Fruity	0.11	0.35	0.54	0.82	0.85	0.23	0.32	0.47	0.56	0.78
2-ethyl-1- hexanol	270	Fruity, ethereal	Fruity	0.00	0.00	0.00	0.00	0.00	0.00	0.00	0.00	0.00	0.00
nonanal	1	Aldehyde,citrus, orange peel	Citrusy	0.05	0.19	0.47	0.65	0.64	0.07	0.01	0.22	0.11	0.06
(E)-2-nonenal	0.08	Cucumber, green	Green	71.01	243.95	115.71	22.46	21.88	18.57	8.45	17.88	30.59	37.10
(E,E)-2,6-nonadienal	0.02	Green,fresh, cucumber	Green, fruity	0.00	34.00	69.50	93.67	157.50	21.50	43.50	84.50	94.14	95.10
1-nonanol	34	Fruity, soapy	Fruity	0.00	0.00	0.00	0.00	0.00	0.00	0.00	0.00	0.00	0.00

Table S4. Odour thresholds (OT), odour description, Odour activity values (OAVs) of green leaf volatiles (GLVs) in Sauvignon Blanc at different stages of the berry maturation in vintage 2019 and vintage 2020.

Compounds	OT[a] (ppb in water)	Odour description	Aromatic series	2019 Sauvignon Blanc OAV[b]					2020 Sauvignon Blanc OAV[b]				
				E-L 34	E-L35	E-L36	E-L 37	E-L 38	E-L 34	E-L 35	E-L 36	E-L 37	E-L38
hexanal	4.5	Green, grassy, fruity	Green, fruity	1.75	15.50	19.43	20.76	30.06	0.00	17.53	29.49	64.30	72.29
3-hexenal	0.21	Green, grassy	Green	0.00	23.84	114.02	7.97	4.86	0.00	0.00	58.70	55.76	53.66
(E)-2-hexenal	17	Green, grassy	Green	4.22	5.49	6.14	3.47	5.09	0.33	3.57	1.14	7.66	7.83
(E,E)-2,4-hexadienal	10	Green, sweet, fruity	Green, fruity	0.00	0.00	0.00	0.00	0.00	0.00	0.00	0.00	0.00	0.00
1-hexanol	150	Green, fruity, flowery, grassy	Green, fruity	5.17	6.48	8.92	7.98	6.27	0.38	0.67	2.04	4.59	4.48
(Z)-3-hexen-1-ol	13	Green,lettuce-like, grassy	Green	0.12	0.10	0.17	0.09	0.04	0.65	0.48	0.08	0.12	0.07
(E)-2-hexen-1-ol	100	Fruity,ethereal	Fruity	0.84	6.07	2.41	2.19	7.94	0.58	2.52	1.17	0.94	1.20
(Z)-3-hexenyl acetate	8	Green; banana-like	Fruity	0.16	0.53	0.92	0.64	0.76	0.17	0.33	0.57	1.03	0.97
2-ethyl-1- hexanol	270	Fruity, ethereal	Fruity	0.00	0.00	0.00	0.00	0.00	0.00	0.00	0.00	0.00	0.00
nonanal	1	Aldehyde,citrus, orange peel	Citrusy	1.40	1.13	1.68	2.80	2.51	0.00	0.24	0.81	1.76	3.26
(E)-2-nonenal	0.08	Cucumber, green	Green	88.15	32.06	29.96	31.65	61.88	20.58	21.81	74.47	73.69	82.78
(E,E)-2,6-nonadienal	0.02	Green,fresh, cucumber	Green, fruity	105.57	97.39	107.38	97.06	172.00	0.00	1.00	102.79	106.67	49.91
1-nonanol	34	Fruity, soapy	Fruity	0.00	0.00	0.00	0.00	0.00	0.00	0.00	0.00	0.00	0.00

Table S5. Odour thresholds (OT), odour description, Odour activity values (OAVs) of green leaf volatiles (GLVs) in Cabernet Sauvignon at different stages of the berry maturation in vintage 2019 and vintage 2020.

Compounds	OT[a] (ppb in water)	Odour description	Aromatic series	2019 Cabernet Sauvignon OAV[b]					2020 Cabernet Sauvignon OAV[b]				
				E-L 34	E-L35	E-L36	E-L 37	E-L 38	E-L 34	E-L 35	E-L 36	E-L 37	E-L 38
hexanal	4.5	Green, grassy, fruity	Green, fruity	0.00	2.30	6.34	6.43	8.43	5.41	12.96	32.00	27.47	38.17
3-hexenal	0.21	Green, grassy	Green	0.00	33.90	42.76	57.67	46.86	0.00	0.00	0.00	3.43	19.72
(E)-2-hexenal	17	Green, grassy	Green	6.66	6.76	12.19	8.81	10.44	0.24	0.28	1.93	6.76	7.29
(E,E)-2,4-hexadienal	10	Green, sweet, fruity	Green, fruity	0.00	0.02	0.03	0.02	0.01	0.00	0.01	0.02	0.02	0.01
1-hexanol	150	Green, fruity, flowery, grassy	Green, fruity	3.56	5.90	4.51	2.70	2.24	0.25	0.41	2.47	2.75	4.73
(Z)-3-hexen-1-ol	13	Green,lettuce-like, grassy	Green	0.06	0.31	0.42	0.26	0.08	0.04	0.15	0.05	0.19	0.13
(E)-2-hexen-1-ol	100	Fruity,ethereal	Fruity	2.81	10.93	6.43	8.40	9.36	0.72	2.73	2.47	3.13	6.29
(Z)-3-hexenyl acetate	8	Green; banana-like	Fruity	0.18	0.17	0.41	0.50	0.58	0.24	0.18	0.18	0.55	0.28
2-ethyl-1- hexanol	270	Fruity, ethereal	Fruity	0.00	0.00	0.00	0.00	0.00	0.00	0.00	0.00	0.00	0.00
nonanal	1	Aldehyde,citrus, orange peel	Citrusy	0.00	0.43	0.52	0.37	0.08	0.00	0.16	0.30	0.19	0.09
(E)-2-nonenal	0.08	Cucumber, green	Green	32.09	30.63	0.00	30.84	93.59	8.63	8.65	9.79	27.13	46.75
(E,E)-2,6-nonadienal	0.02	Green,fresh, cucumber	Green, fruity	0.00	0.00	43.00	98.17	111.12	0.00	0.00	0.00	94.47	96.95
1-nonanol	34	Fruity, soapy	Fruity	0.00	0.00	0.00	0.00	0.00	0.00	0.00	0.00	0.00	0.00

Table S6. Odour thresholds (OT), odour description, Odour activity values (OAVs) of green leaf volatiles (GLVs) in Cabernet Franc at different stages of the berry maturation in vintage 2019 and vintage 2020.

Compounds	OT[a] (ppb in water)	Odour description	Aromatic series	2019 Cabernet Franc OAV[b]					2020 Cabernet Franc OAV[b]				
				E-L 34	E-L35	E-L36	E-L 37	E-L 38	E-L 34	E-L 35	E-L 36	E-L 37	E-L 38
hexanal	4.5	Green, grassy, fruity	Green, fruity	0.00	1.67	8.21	9.51	4.14	0.96	6.36	21.93	27.87	39.94
3-hexenal	0.21	Green, grassy	Green	0.00	0.00	47.08	33.05	10.38	0.00	0.00	0.00	2.19	42.31
(E)-2-hexenal	17	Green, grassy	Green	4.25	5.67	12.39	11.92	6.23	0.40	1.44	3.83	5.89	4.97
(E,E)-2,4-hexadienal	10	Green, sweet, fruity	Green, fruity	0.00	0.01	0.01	0.00	0.00	0.00	0.00	0.01	0.00	0.00
1-hexanol	150	Green, fruity, flowery, grassy	Green, fruity	0.00	0.00	0.04	0.79	1.56	0.00	0.00	0.47	1.93	3.48
(Z)-3-hexen-1-ol	13	Green,lettuce-like, grassy	Green	0.03	0.03	0.05	0.05	0.04	0.08	0.03	0.04	0.32	0.06
(E)-2-hexen-1-ol	100	Fruity,ethereal	Fruity	1.04	3.18	2.97	5.54	6.22	0.65	0.60	0.76	6.11	6.30
(Z)-3-hexenyl acetate	8	Green; banana-like	Fruity	0.16	0.39	0.58	0.55	0.64	0.24	0.36	0.34	0.49	0.41
2-ethyl-1- hexanol	270	Fruity, ethereal	Fruity	0.00	0.00	0.00	0.00	0.00	0.00	0.00	0.00	0.00	0.00
nonanal	1	Aldehyde,citrus, orange peel	Citrusy	0.05	0.16	0.33	0.47	0.30	0.04	0.29	0.68	0.69	0.51
(E)-2-nonenal	0.08	Cucumber, green	Green	57.34	21.94	11.48	19.17	48.99	18.22	29.38	20.20	31.91	38.38
(E,E)-2,6-nonadienal	0.02	Green,fresh, cucumber	Green, fruity	0.00	0.00	0.00	46.87	103.78	0.00	0.00	0.00	95.06	95.99
1-nonanol	34	Fruity, soapy	Fruity	0.00	0.00	0.00	0.00	0.00	0.00	0.00	0.00	0.00	0.00

Table S7. Odour thresholds (OT), odour description, Odour activity values (OAVs) of green leaf volatiles (GLVs) in Cabernet Gernischt at different stages of the berry maturation in vintage 2019 and vintage 2020.

Compounds	OT[a] (ppb in water)	Odour description	Aromatic series	2019 Cabernet Gernischt OAV[b]					2020 Cabernet Gernischt OAV[b]				
				E-L 34	E-L35	E-L36	E-L 37	E-L 38	E-L 34	E-L 35	E-L 36	E-L 37	E-L 38
hexanal	4.5	Green, grassy, fruity	Green, fruity	0.00	1.19	3.86	3.58	4.21	3.63	5.71	16.77	24.64	33.69
3-hexenal	0.21	Green, grassy	Green	0.00	0.00	0.00	0.00	0.00	0.00	0.00	0.00	1.00	0.67
(E)-2-hexenal	17	Green, grassy	Green	0.38	4.46	7.63	6.88	4.86	0.12	1.41	3.15	5.03	4.40
(E,E)-2,4-hexadienal	10	Green, sweet, fruity	Green, fruity	0.00	0.00	0.00	0.00	0.00	0.00	0.00	0.00	0.00	0.00
1-hexanol	150	Green, fruity, flowery, grassy	Green, fruity	0.05	0.36	0.23	1.14	2.54	0.00	0.03	1.05	1.10	2.64
(Z)-3-hexen-1-ol	13	Green,lettuce-like, grassy	Green	0.15	1.46	0.76	0.51	1.55	0.14	0.33	0.17	0.41	0.25
(E)-2-hexen-1-ol	100	Fruity,ethereal	Fruity	5.76	13.04	20.80	13.62	22.22	1.06	1.89	2.35	8.61	18.65
(Z)-3-hexenyl acetate	8	Green; banana-like	Fruity	0.20	0.45	0.55	0.57	0.56	0.37	0.80	0.61	0.52	0.62
2-ethyl-1- hexanol	270	Fruity, ethereal	Fruity	0.00	0.00	0.00	0.00	0.00	0.00	0.00	0.00	0.00	0.00
nonanal	1	Aldehyde,citrus, orange peel	Citrusy	1.25	0.87	1.50	6.53	3.07	0.28	0.94	1.42	3.33	4.89
(E)-2-nonenal	0.08	Cucumber, green	Green	13.24	101.80	16.24	23.55	35.90	17.94	14.35	28.77	19.30	13.69
(E,E)-2,6-nonadienal	0.02	Green,fresh, cucumber	Green, fruity	0.00	0.00	95.25	97.00	101.14	0.00	0.00	0.00	0.00	95.43
1-nonanol	34	Fruity, soapy	Fruity	0.00	0.00	0.00	0.00	0.00	0.00	0.00	0.00	0.00	0.00

Table S8. Odour thresholds (OT), odour description, Odour activity values (OAVs) of green leaf volatiles (GLVs) in Maselan at different stages of the berry maturation in vintage 2019 and vintage 2020.

Compounds	OT[a] (ppb in water)	Odour description	Aromatic series	2019 Maselan OAV[b]					2020 Maselan OAV[b]				
				E-L 34	E-L35	E-L36	E-L 37	E-L 38	E-L 34	E-L 35	E-L 36	E-L 37	E-L 38
hexanal	4.5	Green, grassy, fruity	Green, fruity	4.99	8.75	6.70	7.48	15.18	1.60	2.57	8.35	13.31	12.01
3-hexenal	0.21	Green, grassy	Green	3.39	74.19	43.00	34.09	68.33	0.00	4.62	14.99	69.72	74.03
(E)-2-hexenal	17	Green, grassy	Green	1.18	2.48	4.29	5.49	5.07	0.26	0.34	4.34	4.72	6.10
(E,E)-2,4-hexadienal	10	Green, sweet, fruity	Green, fruity	0.00	0.01	0.02	0.01	0.01	0.00	0.01	0.00	0.00	0.01
1-hexanol	150	Green, fruity, flowery, grassy	Green, fruity	0.19	0.51	0.81	1.71	4.33	0.00	0.00	0.83	1.79	3.25
(Z)-3-hexen-1-ol	13	Green,lettuce-like, grassy	Green	0.04	0.14	0.66	0.15	0.21	0.08	0.07	0.06	0.22	0.09
(E)-2-hexen-1-ol	100	Fruity,ethereal	Fruity	2.75	2.96	5.70	8.02	4.13	0.61	0.79	2.52	7.45	4.34
(Z)-3-hexenyl acetate	8	Green; banana-like	Fruity	0.05	0.18	0.30	0.56	0.74	0.11	0.40	0.59	0.50	0.73
2-ethyl-1- hexanol	270	Fruity, ethereal	Fruity	0.00	0.00	0.00	0.00	0.00	0.00	0.00	0.00	0.00	0.00
nonanal	1	Aldehyde,citrus, orange peel	Citrusy	1.21	0.52	0.96	0.59	0.68	0.00	0.71	0.88	1.22	1.18
(E)-2-nonenal	0.08	Cucumber, green	Green	167.39	209.01	73.67	51.30	18.65	10.67	18.50	18.61	26.51	30.36
(E,E)-2,6-nonadienal	0.02	Green,fresh, cucumber	Green, fruity	39.00	0.00	93.00	98.18	0.00	93.00	0.00	0.00	94.02	94.31
1-nonanol	34	Fruity, soapy	Fruity	0.00	0.00	0.00	0.00	0.00	0.00	0.00	0.00	0.00	0.00

Table S9. Quantitative ion, quantitative standards and calibration curves for quantification of volatile compounds

NO	Compound	CAS	RI[1]	Quantitative standards	Calibration curves	R^2
1	2-ethyl-1-hexanol	104767	1488	2-ethyl-1-hexanol	y = 372.02x-0.30	0.976
2	1-nonanol	143088	1622.3	1-nonanol	y = 666.13x＋0.23	0.969
3	nonanal	124196	1394.8	nonanal	y = 760.00x-0.42	0.973
4	(E, E)-2,4-hexadienal	142836	1408.3	(E)-2-hexenal	y = 8238.82x＋11.36	0.996
5	(E)-2-nonenal	18829566	1542.1	(E)-2-nonenal	y = 4338.17x＋1.33	0.977
6	hexanal	66251	1098.8	hexanal	y = 3922.72x-208.67	0.999
7	3-hexanal	4440657	1178.5	(E)-2-hexenal	y = 8238.82x＋11.36	0.996
8	(E)-2-hexenal	6728263	1204	(E)-2-hexenal	y = 5828.38x-380.83	0.993
9	(Z)-3-hexen-1-ol acetate	3681718	1307.7	ethyl hexanoate	y = 1991.68x-0.14	0.984
10	1-hexanol	111273	1347.1	1-hexanol	y = 1636.43x＋0.00	0.999
11	(Z)-3-hexen-1-ol	928961	1381	(Z)-3-hexenol	y = 7898.50x＋35.81	0.99
12	(E)-2-hexen-1-ol	928950	1403.4	(E)-2-hexenol	y = 2685.83x＋0.00	0.999
13	(E, E)-2,6-nonadienal	17587336	1594.6	(E)-2-nonenal	y = 1766.26x＋2.11	0.983

Notes:

a. Retention indices were calculated on HP-INNOWAX column.

b. The concentration of these compounds expressed as relative areas (to 4-methyl-2-pentanol).

Table S10. Primer used in real-time PCR

Gene	Sense	Antisense
VvActin	GCATCCCTCAGCACCTTCCAGCAG	CCACCTCAACACATCTCCATGTCAACC
VvLOXA	GCAAATCAAAGGGACAACGCTGTATG	TGCTTCCACTGCGGCTTCC
VvHPL	AAGTACACCGGCGACATTCGAG	AGCTCTTTACCCTGGCGTGTTG
VvADH1	TCCGTTCTCAGAGATCAACAA	ACTCTCTCATCTCAAGATATTCTATGG
VvADH2	ATTCCAGTCGGCATAAGTGT	TTGCAACTGCATAGACATTGTT
VvAAT	TTAATTCAGGTGAcCCGATT	TCTCCATACACATGCCATTAG

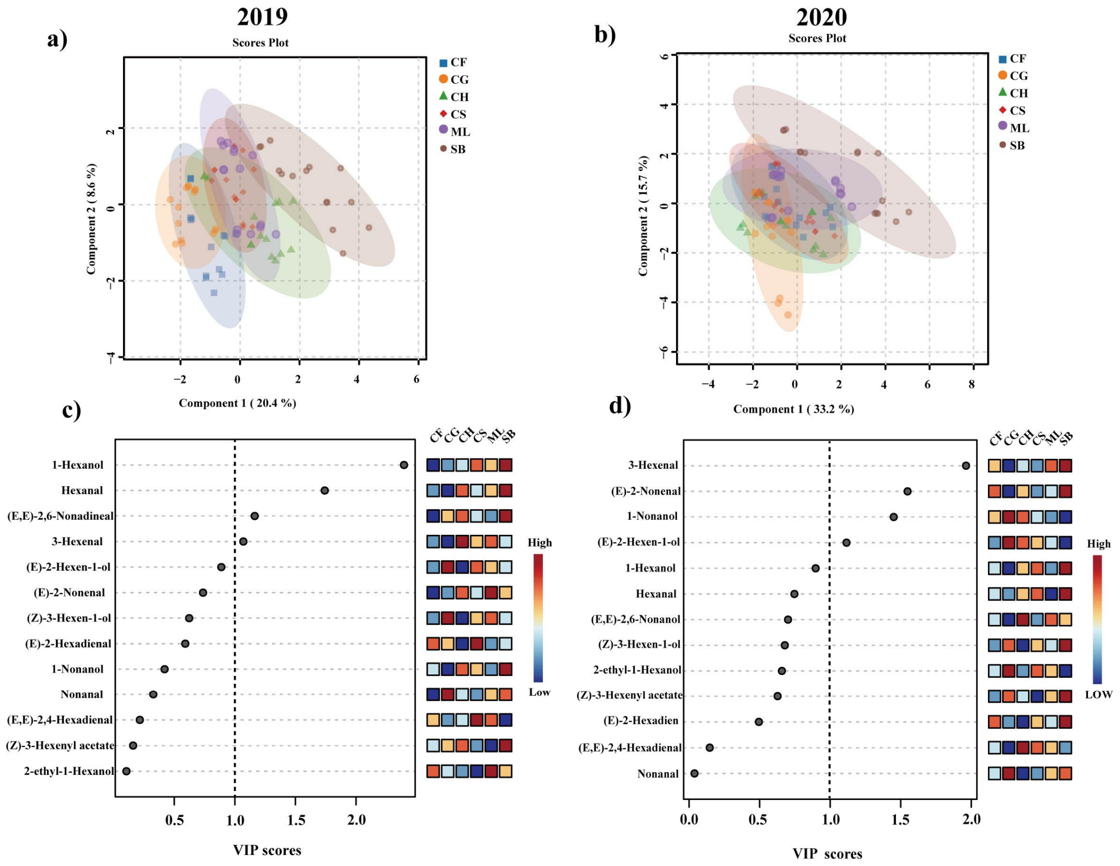

Fig. S1. Partial Least Squares Discriminant Analysis biplot illustrating the pattern of green leaf volatiles (GLVs) at different stages of the berry maturation in vintage 2019 (a) and vintage 2020 (b). VIP diagrams illustrate key differential compounds in vintage 2019 (c) and vintage 2020 (d). Legend: ▲-Chardonnay, ●-Sauvignon Blanc, ◆-Cabernet Sauvignon, ■-Cabernet Franc, ▲-Cabernet Gernischt, ●-Marselan.
